# Imaging‐Based Efficacy Evaluation of Cancer Immunotherapy in Engineered Tumor Platforms and Tumor Organoids

**DOI:** 10.1002/adhm.202400475

**Published:** 2024-06-08

**Authors:** Seong‐Eun Kim, Suji Yun, Junsang Doh, Hong Nam Kim

**Affiliations:** ^1^ Brain Science Institute Korea Institute of Science and Technology (KIST) Seoul 02792 South Korea; ^2^ Interdisciplinary Program for Bioengineering Seoul National University Seoul 08826 South Korea; ^3^ Department of Materials Science and Engineering Research Institute of Advanced Materials Institute of Engineering Research Bio‐MAX institute Soft Foundry Institute Seoul National University Seoul 08826 South Korea; ^4^ Division of Bio‐Medical Science and Technology KIST School Korea University of Science and Technology Seoul 02792 Republic of Korea; ^5^ School of Mechanical Engineering Yonsei University Seoul 03722 Republic of Korea; ^6^ Yonsei‐KIST Convergence Research Institute Yonsei University Seoul 03722 Republic of Korea

**Keywords:** bioimaging, cancer immunotherapy, efficacy evaluation, engineered tumor platform, tumor organoid

## Abstract

Cancer immunotherapy is used to treat tumors by modulating the immune system. Although the anticancer efficacy of cancer immunotherapy has been evaluated prior to clinical trials, conventional in vivo animal and endpoint models inadequately replicate the intricate process of tumor elimination and reflect human‐specific immune systems. Therefore, more sophisticated models that mimic the complex tumor‐immune microenvironment must be employed to assess the effectiveness of immunotherapy. Additionally, using real‐time imaging technology, a step‐by‐step evaluation can be applied, allowing for a more precise assessment of treatment efficacy. Here, an overview of the various imaging‐based evaluation platforms recently developed for cancer immunotherapeutic applications is presented. Specifically, a fundamental technique is discussed for stably observing immune cell‐based tumor cell killing using direct imaging, a microwell that reproduces a confined space for spatial observation, a droplet assay that facilitates cell–cell interactions, and a 3D microphysiological system that reconstructs the vascular environment. Furthermore, it is suggested that future evaluation platforms pursue more human‐like immune systems.

## Graphical abstract

The imaging‐based cancer immunotherapy evaluation platform facilitates systematic assessment of treatments through suitable in vitro models that closely mimic the human immune system. These evaluation methods enable a more precise and personalized approach in the development of cancer immunotherapy.

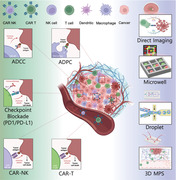

## 1 Introduction

Cancer immunotherapy utilizes the patient's immune system to eliminate tumors by restoring or regenerating the immune response. Immune checkpoint blockade (ICB) activates T cells by blocking inhibitory receptor–ligand interactions of cytotoxic T‐lymphocyte antigen 4 (CTLA‐4) and programmed cell death protein 1 (PD‐1). With advances in genetic engineering, adoptive cell therapy (ACT), which involves infusing ex vivo engineered cytotoxic lymphocytes (CLs) into patients, has been used clinically to lyse tumors.^[^ ^1^ ^]^ In particular, chimeric antigen receptor (CAR) T‐cell therapies targeting CD19 and B cell maturation antigen (BCMA) have been approved by the US Food and Drug Administration for the treatment of B‐cell malignancies.^[^ ^2^, ^3^ ^]^ CAR‐T cells, CAR‐natural killer (NK) cells, and CAR‐macrophages are currently undergoing clinical trials to treat solid tumors.^[^ ^4^ ^]^

Given the diversity and complexity of immune cell therapy candidates, reproducible high‐throughput screening assays are crucial to effectively select the most suitable therapeutics. The initial screening stage involves assessing the capacity of effector cells to induce cytotoxicity against tumors. Despite an enhanced understanding and encouraging clinical outcomes, several challenges remain with contemporary immunotherapeutic approaches. These challenges involve thoroughly assessing cytotoxicity in preclinical investigations with combinations of immunotherapies,^[^ ^5^ ^]^ comprehending the molecular and cellular catalysts driving immune cell migration and immune–tumor cell interactions,^[^ ^6^ ^]^ and optimizing personalized medicines. Consequently, preclinical testing models are being developed to replicate human immunity and the tumor immune microenvironment (TIME).^[^ ^7^ ^]^

Alternative 3D models present novel prospects for scrutinizing intricate interactions that occur within biological microenvironments under normal and pathological conditions.^[^ ^8^, ^9^ ^]^ Although 2D human cell cultures offer valuable, simple, and easy access to human biology, they fail to adequately represent the complexity and diversity of in vivo tissues.^[^ ^10^ ^]^ The cell–cell and cell–environment interactions in 2D cell cultures differ from those found in natural tissues. Tumor cells cultured in 2D systems exhibit lower resistance to chemotherapy than those cultured in 3D systems, which can potentially lead to misleading outcomes in drug screening.^[^ ^11^ ^]^ In contrast, 3D culture systems can more accurately recapitulate cell–cell interactions, matrix deposition, cell microenvironments, and physiological conditions such as flow, oxygen, and nutrient gradients.^[^ ^12^, ^13^ ^]^ These 3D cell culture models are promising as they serve as a valuable bridge between 2D culture and animal models. Consequently, they have attracted substantial interest in various fields ranging from fundamental research in cancer and stem cell biology to applications in drug toxicity testing and high‐throughput screening. Moreover, these 3D models are promising for advancing personalized medicine and next‐generation drug screening, while reducing reliance on animal experimentation.^[^ ^14^ ^]^

In this review, we comprehensively discuss the efficacy of cancer immunotherapy from standard cytotoxicity assays to next‐generation image‐based cytotoxicity assays. First, we elaborate on the principles, methodologies, and applications of these assays, while carefully evaluating their individual strengths and weaknesses. We aimed to demonstrate the efficacy and limitations of these assays for assessing cytotoxic effects. Furthermore, we discuss specific parameters that can be evaluated using each assay, including TIME, immune synapses (IS), cell heterogeneity, and target cell culture methods. We also review recent advances in image‐based analysis methods for cancer immunotherapy, including direct real‐time observation, microwell‐based confinement, droplet‐based cell–cell interactions, and 3D microphysiological systems (MPS)‐based TIME modeling. Finally, we describe the need to develop more physiologically relevant testing platforms and image‐based analysis methods for cancer immunotherapy. Image‐based analyses of human‐immune system‐mimetic models can facilitate the development of cancer immunotherapeutics.

## 2 Cancer Immunotherapy

Cancer immunotherapy aims to eradicate tumors by harnessing the immune system. Antitumor immune responses comprise diverse mechanisms, such as infiltration into tumor tissues and the identification and killing of tumor cells. Various types of CLs, including cytotoxic T lymphocytes (CTLs), NK cells, and γδ T cells, act as key players in cancer immunotherapy by directly eliminating cancers.^[^ ^15^, ^16^, ^17^, ^18^, ^19^ ^]^ Antitumor immune responses occur cyclically, known as the cancer‐immune cycle, and involve dynamic cellular processes that span tumor tissues and lymph nodes. The process was divided into seven stages.

1. Release of cancer cell antigens through cancer cell death
2. Presentation of cancer antigen by dendritic cells (DCs)
3. Priming and activation of tumor‐reactive T cells by DCs
4. Trafficking of T cells to tumor tissues via blood vessels
5. Infiltration of T cells into tumors
6. Recognition of cancer cells by T cells
7. Killing of cancer cells by T cells^[^ ^20^ ^]^

Enhancing the cytotoxic function of T cells is crucial because T cell‐mediated killing of cancer cells promotes the release of cancer antigens, thereby amplifying the overall immune response. Consequently, immunotherapies have been developed to enhance the tumor‐killing ability of immune cells. To counter the various immunosuppressive challenges posed by the TIME and facilitate tumor eradication, either the antitumor functions of immune cells in cancer patients are boosted, or patients are administered immune cells showing enhanced functions that were engineered ex vivo.^[^ ^21^ ^]^ This is achieved through a multifaceted process involving extravasation, which is critical for immune cell infiltration into tumor tissues and immune synapse formation, which mediates tumor cells recognition and killing by T cells.

### 2.1 Typical Cases of Cancer Immunotherapy

ACT, which involves the infusion of ex vivo engineered CLs into patients, has emerged as a promising approach for treating various types of tumors. The Rosenberg group pioneered ACT based on the ex vivo expansion of tumor‐reactive T cells enriched with tumor‐infiltrating lymphocytes (TILs).^[^ ^22^ ^]^ However, the isolation of tumor‐reactive T cells requires a long preparation time and has a low success rate. To address this issue, the Rosenberg group cloned T‐cell receptor (TCR) genes specific for well‐known tumor antigens and transferred them into normal T cells to develop TCR‐T therapy.^[^ ^23^ ^]^ Although the initial clinical results were promising, the applicability of TCR‐T‐cell therapy was limited to patients expressing specific human leukocyte antigen (HLA) types because of HLA‐dependent antigen recognition by TCR. CAR T‐cell therapy has been developed to overcome these limitations. CAR‐T cells express CAR, a chimeric protein that combines the extracellular portion of a highly specific antibody single‐chain variable fragment (scFv) targeting a cancer antigen, with an intracellular signaling chain derived from TCR signaling units. Unlike TCR T‐cell therapy, CAR T‐cell therapy can be used in all patients regardless of HLA variability. This breakthrough has been successful in treating CD19‐expressing leukemia and lymphoma^[^ ^24^, ^25^ ^]^ and BCMA‐expressing multiple myeloma.^[^ ^26^, ^27^, ^28^ ^]^

ICBs are another approach to cancer immunotherapy that is widely used in clinics. ICBs block receptors that inhibit T‐cell activation (such as CTLA‐4 and PD‐1).^[^ ^29^, ^30^, ^31^ ^]^ The inhibition of CTLA‐4 or PD‐1 signaling in T cells has demonstrated remarkable efficacy in patients with various tumors, including melanoma and lung cancer. In particular, the applications of anti‐PD‐1/L1 ICB therapies are expanding in combination with other drugs (doxorubicin, cisplatin, trastuzumab, bevacizumab, etc.).^[^ ^32^, ^33^, ^34^ ^]^

Despite the great success of CAR‐T‐cell therapy and ICBs, the development of novel cancer immunotherapeutic agents continues to widen their applicability and minimize their side effects. To enhance the therapeutic efficacy, a comprehensive understanding of the entire process, from antigen presentation to tumor eradication, is essential. This comprehensive insight allows for rapid feedback on the stage at which new immunotherapeutic agents encounter challenges and can guide the trajectory toward individualized patient treatment.

### 2.2 TIME

Despite using the same treatment method, variations in treatment responses were observed among different patients. A retrospective analysis of patients treated with ICBs revealed an association between TIME class and responsiveness to ICBs.^[^ ^35^ ^]^ T cells are activated in lymph nodes or ex vivo‐engineered immune cells, enter blood vessels, and navigate through the circulatory system to eventually exit the blood vessels near the malignant tissue or tumor stroma. Extravasation involves a leukocyte adhesion cascade.^[^ ^36^ ^]^ Briefly, immune cells undergo transient tethering and rolling interactions with the endothelium mediated by selectins. This facilitates the sensing of chemokines present on the inflamed endothelium, leading to increased cell–cell contact and the activation of lymphocyte integrins by mechanotransduction. Consequently, immune cells firmly adhere to the inflamed endothelium and actively crawl around to locate exit sites. After extravasation, immune cells migrate to the tumor stroma, which is densely packed with the extracellular matrices (ECMs). The biophysical and biochemical properties of the tumor ECM are crucial for regulating immune cell migration and infiltration for direct contact with tumor cells.^[^ ^37^, ^38^, ^39^, ^40^ ^]^ ECM consists of fibrous proteins (such as hyaluronic acid, proteoglycans, collagens, and fibronectin) and stromal cells (fibroblasts). Collagen and elastin, the major structural fibers, contribute to the ECM stiffness and provide mechanical support. The migration patterns of macrophages and T cells depend on collagen expression.^[^ ^41^, ^42^, ^43^, ^44^ ^]^ Elastin also facilitates recruitment of mononuclear phagocytes and is crucial for macrophage migration.^[^ ^45^, ^46^ ^]^ Cancer‐associated fibroblasts (CAFs) are activated by signaling molecules such as transforming growth factor‐beta, platelet‐derived growth factor, and hepatocyte growth factor secreted from cancer cells. CAFs remold the ECM, which becomes resistant to degradation by matrix metalloproteinases (MMPs) owing to intermolecular crosslinks between type 1 collagen, thereby enhancing the rigidity of the matrix. Both tumor cells and CAFs secrete vascular endothelial growth factor, which results in the formation of abnormal and dysfunctional tumor vasculature, further exacerbating the tumor microenvironment (TME).^[^ ^47^ ^]^ Various factors mentioned above, such as inhibitory molecules expressed by tumor cells, ECM altered by CAFs, and abnormal vasculature, induce the desensitization and debilitation of TILs. Consequently, TILs exhibit reduced efficacy in evoking potent antitumor immune responses. Hypoxia, resulting from diminished oxygen levels and acidity, occurs within the TME and is primarily caused by tumor vascular leakage and compression. Under these adverse circumstances, the TME considerably affects both tissue‐resident and circulating immune cells in the bloodstream, culminating in immunosuppression. Specifically, upregulation of hypoxia‐inducible factor alpha promotes the enhancement of immunosuppressive activity in myeloid‐derived suppressor cells (MDSCs) and tumor‐associated macrophages (TAMs). Moreover, hypoxia‐inducible factors primarily exert negative regulatory effects, including inhibition of effector functions within TILs.^[^ ^48^ ^]^

Different subclasses of the immune environment influence tumor initiation and response to therapy. Therefore, understanding the characteristics of TIME in a patient's tumor can potentially guide the development of personalized treatment strategies.^[^ ^49^ ^]^

### 2.3 Dynamic Immune Synapse Formation for Recognizing and Killing Tumor Cells

As immune cells contact cells in the tumor tissue after extravasation and interstitial migration through the tumor stroma, they initiate dynamic cell–cell interactions and form IS.^[^ ^50^ ^]^ The initial IS shows transient and dynamic cell–cell contacts. Different types of CLs utilize various antigen‐recognizing and activating/inhibitory receptors to integrate signals from target cells through IS to determine whether they continue to interact with or migrate to other cells.^[^ ^51^ ^]^

In the effector phases of the IS, polarization of the cytoskeleton and effect molecules on the IS occur.^[^ ^52^ ^]^ First, F‐actin accumulation in the IS flattened and stabilized cell‐cell interfaces with adhesion receptor‐mediated firm adhesion. The microtubule‐organizing center and lytic granules, which contain cytotoxic molecules such as perforin and granzyme B, are polarized to the IS.^[^ ^53^, ^54^ ^]^ Finally, exocytosis of lytic granules toward the IS releases perforin, which forms pores in target cell membranes, and granzyme, which induces apoptosis of target cells by triggering caspase‐dependent death pathways.^[^ ^55^ ^]^ The final step in the cycle is the detachment of CLs from dying/dead target cells to engage with another target. The detachment step is critical for serial killing of tumor cells, and CLs that fail to detach from dead targets may undergo activation‐induced cell death.^[^ ^56^ ^]^

Single‐cell‐level analysis of immune cell–cancer cell interactions using live‐cell imaging revealed substantial functional heterogeneity in immune cell cytotoxicity, even for CLs cultured in the same batch. For example, a small fraction of NK cells exhibited serial killing, whereas most NK cells did not exhibit cytotoxicity throughout the experiment.^[^ ^57^ ^]^ Therefore, the functions of ex vivo‐engineered immune cells for ACT may require single‐cell‐level analysis to account for the heterogeneity in their functions.

### 2.4 Cancer Cells in 2D Versus 3D

**Figure 1:**
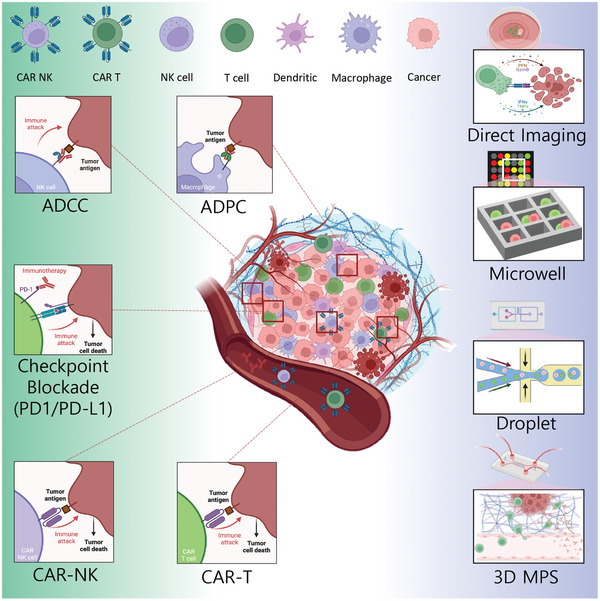
Schematic illustration of microfabricated platforms recapitulating diverse tumor‐immune microenvironments. The figure illustrates the tumor microenvironment of a solid tumor, which comprises diverse immune cells. Proinflammatory immune cells actively infiltrate into the tumors and kill them. Through various cancer immunotherapies, these infiltrated immune cells attach to tumor cells and form an immune synapse which facilitates the recognition of the target cell and activation of the effector functions toward the tumor (left panel). Various cytotoxicity evaluation assays based on imaging methods, including direct imaging, microwell assay, droplet assay, and 3D MPS chips (right panel). Images were created using BioRender.com.

In vitro studies of immune cell–tumor cell interactions must be carefully interpreted, as they are mainly performed in artificial 2D culture systems. Experimental models that faithfully reproduce the TIME are required to fully understand immune cell–cancer cell interactions in the TIME.^[^ ^58^ ^]^ The 2D models rely on the adherence of cells to a flat culture platform and provide unlimited access to nutrients and oxygen in the culture medium. Although this model can be used in preliminary investigations to evaluate the direct effects of biophysical and biochemical cues on tumors, it does not mimic the natural structure of tumors. Recent studies have focused on culture platforms with 3D structures and more realistic biochemical and biomechanical microenvironments. An example of a 3D cell culture platform, tumor spheroid or tumor‐organoid culture, is being created to better mimic in vivo conditions.^[^ ^59^, ^60^, ^61^, ^62^ ^]^ 3D spheroid culture methods include microfluidics,^[^ ^63^ ^]^ microchips,^[^ ^64^ ^]^ embryoid bodies, collagen gels,^[^ ^65^ ^]^ and hanging drop culture.^[^ ^66^, ^67^, ^68^ ^]^ Tumor organoids have a significant impact on cancer immunotherapy by providing patient‐specific 3D models.^[^ ^69^, ^70^, ^71^, ^72^, ^73^ ^]^ In vitro models suitable for studying the interactions between tumor organoids and immune cells have been reported, including gastric cancer organoids with CD8+ T cells and DCs,^[^ ^74^ ^]^ rectal cancer with autologous TILs,^[^ ^75^ ^]^ and pancreatic cancer with allogeneic T cells^[^ ^76^ ^]^ (**Figure** **1**).

## 3 Conventional Cytotoxicity Assay

Despite substantial advancements in immunotherapy and promising clinical results, several challenges remain when evaluating the efficacy of cancer immunotherapy. These challenges include a deeper understanding of the molecular mechanisms involved in immune cell migration and immune–tumor interactions and the optimization of personalized medicine approaches. Preclinical studies are essential to accelerate the development of new CLs and to evaluate their efficacy. Given the diverse and complex nature of immunotherapy, reproducible and high‐throughput screening assays are required to assess therapeutic effectiveness. Cytotoxicity assays measuring effector cell‐mediated tumor cell killing are commonly used to screen therapeutics. In this section, we describe a standard assay for quantifying the target cell lysis mediated by effector cells. We focused on four commonly employed assays that differed in terms of target cell labeling, coincubation time, primary measure of cytotoxicity, and number of measurements (transient or endpoint). Furthermore, we explained the underlying principles of these assays and discussed their advantages and limitations. The key information regarding the assay methods is summarized in **Table** **1**.

**Table 1:** Summary of representative methods for cytotoxicity assay.

Type of cytotoxicity assay	Advantages	Disadvantages	Cellular heterogeneity	Detection time point	High‐throughput	TIME^a)^ reflection	Ref.
Release assay	Simple, low cost, possibility of combining with other read‐outs in a single test run	Spontaneous leakage, unstable signals, limited dye uptake in certain cell types	Population level	End point	Yes	No	[83, 84, 85, 86, 87]
Flow cytometry	Measurement of single cells or a large number of cells, multiparameter analysis, sorting	Expensive instruments, highly skilled and experienced labor	Population level	End point	No	No	[96, 97, 98, 99, 100, 101]
Mouse model	Whole organism level system	Time‐consuming, high costs, ethical concerns, human‐mouse immune system mismatch	Population level	End point	No	Yes	[103, 104, 105, 106, 107]
Direct imaging	Detailed information on cell–cell interactions	Lack of 3D microenvironment, time‐consuming data collection and analysis	Single cell level	Real‐time	No	No	[113, 115, 116]
Microwell	Simple fabrication, quantitative analysis for cell heterogeneity	Lack of 3D microenvironment, difficult to isolate cells after analysis	Single cell level	Real‐time	Yes	No	[121, 122, 123, 124, 125, 126]
Droplet microfluidics	Possibility to isolate cells for further analysis, well‐controlled cell to cell pairing, small sample volume	Lack of 3D microenvironment, random encapsulation (follow Poisson distribution)	Single cell level	Real‐time	Yes	No	[154, 155, 156, 157, 158, 159, 160, 161, 162, 163]
3D MPS	3D microenvironments containing various TIME components fluidic condition	Difficult to isolate cells after analysis, relatively complex fabrication	Single cell level	Real‐time	Yes	Yes	[172, 173, 174, 175, 176, 177, 179, 180, 183, 184]

^a)^ TIME: tumor‐immune microenvironment.

### 3.1 Cr^51^ and Calcein Acetoxymethyl Release Assay

One of the most widely used methods for evaluating immune cell‐mediated cytotoxicity is the Cr^51^ release assay. This method is based on the quantification of tumor cells with compromised membrane integrity. Cancer cells loaded with radioactive chromium were mixed with CLs at various effector‐to‐target cell (E:T) ratios. Over a specific period, CLs lyse cancer cells, causing the release of Cr^51^ into the medium. The radioactivity of the supernatant was then measured. This measurement indicates the extent of target cell death within a designated timeframe. To calculate the percentage of specific lysates, the following control samples were included: a continuous‐release control (containing only the target cells in the culture medium) and a maximum‐release control (containing the target cells in the culture medium with Triton X‐100). The percentage specific lysis was determined using the following equation: [(experimental release − spontaneous release)/(maximum release − spontaneous release)] x 100.^[^ ^77^, ^78^ ^]^

**Figure 2:**
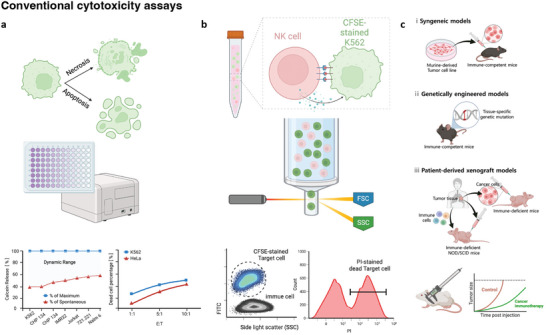
Comparison of cell‐mediated antitumor efficacy evaluation models. a) Calcein‐AM release assay: calcein release of labeled target cells cocultured with effector cells at different effector‐to‐target (E:T) ratios determined relative to a maximum and a spontaneous calcein release control. b) Flow cytometry assay: effector cell‐mediated killing of CFSE‐stained target cells revealed through the PI signal. c) Diverse mouse models for evaluating cancer immunotherapy. Images were created using BioRender.com.

The Cr^51^ release assay is considered the “gold standard” for assessing the killing function of NK cells and cytotoxic T lymphocytes.^[^ ^79^, ^80^, ^81^, ^82^ ^]^ However, alternative methods have been developed because of concerns regarding the toxicity associated with the handling and disposal of radioactive compounds, and the requirement for expensive equipment and specialized staff. An alternative method introduced in 1994 utilizes a fluorescent dye called calcein acetoxymethyl (AM), which operates on a principle similar to that of Cr^51^.^[^ ^83^ ^]^ Calcein AM is known to exhibit variable loading efficiencies in different cell lines and a higher spontaneous release than chromium. The assay sensitivity and dynamic range may be reduced by high spontaneous release and low loading efficiency in certain cell lines.^[^ ^84^ ^]^ Nevertheless, the release assay showed a good correlation with the percentage of specific lysates, leading many research groups to routinely employ it in immune cell cytotoxicity assays (**Figure** **2**A).

A limitation of this method is that errors can occur depending on the type of target cell. During necrosis, the target cell membrane is damaged, which results in the release of calcein into the medium. However, in apoptosis, the target cell forms morphological “blebs” and entrapped calcein may remain within the apoptotic bodies of the lysed target cell without being fully released. This incomplete release of calcein from target cells can result in an underestimation of the actual lysis percentage. Modifications have been implemented to address these limitations, such as a combination of flow cytometry (fluorescence‐activated cell sorter, FACS)^[^ ^85^ ^]^ and image processing technology.^[^ ^86^ ^]^ Additionally, cytotoxicity can be measured by crosschecking with dead markers such as SYTOX or propidium iodide (PI).^[^ ^87^ ^]^

### 3.2 Bioluminescence (BLI) Assay

BLI‐based cytotoxicity assays utilize the emission of light (photons) from engineered target cells to express luciferase reporter genes. The luciferase substrate was specifically processed by live cells carrying a luciferase transgene. Consequently, the cytotoxicity of immune cells could be quantified by reducing the BLI signal.^[^ ^88^ ^]^ Luciferase‐based cytotoxicity assays were performed to measure the activity of firefly luciferase secreted into the supernatant after targeted cell lysis. However, because of the short half‐life (≈30 min) of firefly luciferase in culture medium, these assays were limited to conditions in which substantial target cell lysis occurred within a few hours.^[^ ^89^ ^]^ In addition, the molecular weight of firefly luciferase (62 kDa) may impede its efficient release into the supernatant upon effector cell‐induced pore formation in target cells. Therefore, this method may not be suitable for cytotoxicity studies conducted over prolonged periods.^[^ ^90^, ^91^ ^]^

Both BLI and Cr^51^ assays revealed an E:T ratio‐dependent increase in cytotoxicity. However, the BLI assay offers a higher signal‐to‐background ratio and up to twofold higher percentage of specific lysis than the Cr^51^ Assay. Studies have shown comparable signal‐to‐background ratios between nanoluciferase and Cr assays, with similar cytotoxicity curves, but slight differences in half‐maximum effective concentration (EC50) values, likely due to the different release mechanisms of nanoluciferase and Cr^51^.^[^ ^92^ ^]^ The BLI assay has gained widespread use owing to its ease of handling and simple quantification. However, a prerequisite for BLI‐based reporter assays is luciferase expression in the target cells.^[^ ^93^, ^94^ ^]^ Stable transfection of cells can be time‐consuming and may not be feasible for all cell types. Therefore, the BLI assay is particularly valuable when the same target cell line is used for screening. The low transfection efficiency of plasmid‐based approaches in primary cells can be overcome by electroporating the target cells with luciferase gene‐encoding RNAs generated in large quantities via in vitro transcription.^[^ ^95^ ^]^

### 3.3 Flow Cytometry Assay

Unlike the aforementioned assays, flow cytometric cytotoxicity assays enable the analysis and quantification of functional cell subpopulations within cell mixtures, making them advantageous for investigating the heterogeneity of cell populations.^[^ ^96^ ^]^ This assay distinguishes target and effector cells based on their size and granularity (measured by forward and sideward scatter, respectively) and specific staining with fluorochrome‐conjugated antibodies. Target cells are labeled with carboxyfluorescein diacetate succinimidyl ester (CFSE) dye for 10 min at 37 °C, followed by two washes with complete media to halt the labeling reaction. The stained target cells are coincubated with immune cells at various E:T ratios in a humidified 5% CO_2_ incubator at 37 °C. After a few hours of coincubation, the cell mixture was transferred to an FACS tube, and DNA‐intercalating fluorescent agents such as PI, 7‐aminoactinomycin D, SYTOX, and annexin V were added as cell death markers for evaluating cell death.^[^ ^97^, ^98^ ^]^ The fluorescently labeled cells were analyzed using a flow cytometer and the percentage of dead target cells displaying CFSE‐positive and PI‐positive signals was calculated by subtracting the percentage of spontaneously dead target cells^[^ ^99^ ^]^ (Figure 2B). Reports utilizing either target or effector cell staining in conjunction with cell death or apoptosis markers have shown a positive correlation between Cr and FACS assay.^[^ ^96^, ^100^, ^101^ ^]^ Furthermore, flow cytometry assays can be used to identify the mechanisms of cancer cell death using various antibodies (CD107a, granzyme B, and interferon‐gamma (IFN‐γ)). Packard et al. developed a similar approach for measuring cellular apoptosis based on the activation of intracellular proteases using cell‐permeable fluorescent substrate probes. This method allows quantification of granzyme B entry into target cells as an early event in cell‐mediated cytotoxicity.^[^ ^102^ ^]^ The ability to simultaneously analyze target cell killing and effector cell phenotypes and discriminate heterogeneous cell populations allows the study of the correlation between stimulatory and inhibitory signaling within a single experiment. Experienced professionals have varying opinions regarding gating strategies. In addition, multivariate experiments must be performed repeatedly, and care must be taken when designing and interpreting the results.

### 3.4 Mouse Models

Syngeneic tumor models have been used as preclinical models to evaluate antitumor therapies (Figure 2Ci). These models involve the injection of tumor cell lines that can be expanded in vitro into inbred mouse lines such as C57BL/6 and BALB/c mice. The antitumor efficacy of cancer immunotherapy can be evaluated in tumor‐bearing mice.^[^ ^103^ ^]^ For instance, a tumor cell line can be induced using agents such as methylcholanthrene to facilitate the identification and characterization of the immunoediting process.^[^ ^104^ ^]^ These models offer the advantages of ease of use and experimental reproducibility because tumor cell lines can be rapidly expanded in large quantities before transplantation into the host. However, these models lack the genomic and microenvironmental heterogeneity that is observed in human cancers in vivo. Tumor heterogeneity exists not only between patients, making each cancer unique, but also among patients.^[^ ^105^ ^]^ Genetically engineered mouse models (GEMMs) have emerged with advances in our understanding of the genetic basis of cancer cells and the development of genetic engineering techniques (Figure 2Cii). GEMMs target the expression of viral oncogenes, such as the SV40 large T antigen,^[^ ^106^ ^]^ and oncogenes associated with tumorigenesis, such as Kirsten rat sarcoma virus (KRAS) and MYC, in breast cancer cells.^[^ ^107^ ^]^ These models induce the neoplastic transformation of normal cells in specific organs, allowing for tumor growth and the gradual development of a complex TME. GEMMs offer advantages over syngeneic tumor models for the evaluation of immunotherapeutic modalities. However, unlike human diseases in which mutations gradually accumulate in specific cells, leading to genetic diseases, GEMMs that utilize tissue‐specific promoters induce carcinogenesis in all cells. This may be due to the overexpression of certain genes, resulting in a tumor mutational load that does not fully replicate that observed in corresponding human diseases.^[^ ^108^ ^]^ In recent years, patient‐derived tumor xenograft (PDTX) models have been shown to be superior in recapitulating cancer properties, including the spatial structure and intratumoral heterogeneity of cancer, by transplanting tumor tissues into immunocompromised or humanized mice (Figure 2Ciii). These models have been instrumental in developing CAR therapies and evaluating their antitumor efficacy by using human cell lines or patient‐derived samples to generate xenografts.^[^ ^109^ ^]^ Despite the usefulness of PDTX models in cancer research, some important limitations must be addressed to improve their application in translational cancer research. These limitations include technical issues such as determining the most appropriate tissue for generating a patient‐derived xenograft (PDX) model and optimizing tissue processing. Additionally, PDX models require high uptake rates to successfully propagate tumors across multiple mice within a reasonable time. However, the development of humanized PDX mice requires repeated invasive sampling.

Several research groups have evaluated the efficacy of cancer immunotherapeutic agents using these mouse models. They assessed whether tumor growth was inhibited by measuring tumor size and performing intravital imaging, which allows for high‐resolution observation of biological processes in live animals.^[^ ^110^, ^111^ ^]^ Additionally, some research groups conducted immunohistochemical staining using antibodies that recognize specific target antigens. However, these methods raise concerns regarding time constraints and ethical considerations regarding animal use.

## 4 Microfabricated Platforms for Evaluating Immune Cell–Cancer Cell Interactions in Cancer Immunotherapy

Ex vivo‐engineered CLs undergo a series of crucial steps upon infusion through veins to exert cytotoxicity against solid tumors. Initially, CLs exit the blood vessels near tumor tissues and migrate through interstitial spaces filled with ECMs to reach the tumor site. Subsequently, CLs establish dynamic synapses with tumor cells, recognize distinct molecular signatures, and unleash their cytotoxic potential by exocytosing lytic granules containing cytotoxic molecules to induce cancer cell death. Any defects occurring during these steps can substantially affect overall cytotoxicity, emphasizing the importance of the rational design of engineered CLs.

Furthermore, the interactions between cancer cells and CLs and the process of synapse formation must be analyzed. Recent advancements in real‐time microscopy techniques and live/dead staining methods have led to the development of various in vitro platforms for assessing the efficacy of antitumor treatments.^[^ ^112^ ^]^ Unlike conventional assays that primarily provide population‐level and endpoint information, real‐time imaging allows for the long‐term tracking of individual lymphocytes, enabling a deeper understanding of the intricate steps leading to tumor cell death. In addition, the heterogeneity of the same type of CLs can be analyzed at the single‐cell level.

Microfabrication technology has facilitated the development of chip‐based cytotoxicity assays that better reflect the characteristics of the human immune system. These microfabricated platforms include microwell arrays, droplet arrays, and organ‐on‐a‐chip designed to recapitulate the TIME. By utilizing these advanced platforms, CL trafficking and killing can be assessed in a 3D context, thereby providing valuable insights into the cytotoxic capabilities.

### 4.1 Direct Real‐Time Imaging Assay for Assessing Immune Cell–Cancer Cell Interactions

**Figure 3:**
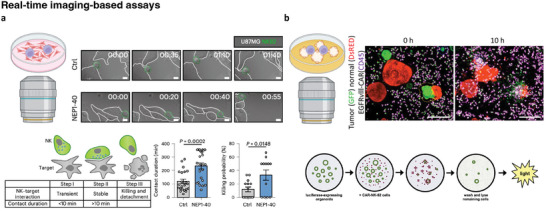
Direct real‐time imaging assay of tumor‐immune cell interaction using live cell imaging technology. Cancer cells are cultured on a dish and a small amount of immune cells are seeded in the dish. Then, the cellular behaviors are observed in real time. a) Live cell imaging of the NK cell killing process in vitro. Representative time‐lapse images of interaction between NK92 (green borderline) and U87MG (white borderline) with and without NEP1–40 treatment. Scale bar: 10 µm (Reproduced with permission.^[^ ^115^ ^]^ Copyright 2023, Springer Nature). b) Live cell imaging of CAR‐mediated cytotoxicity against tumor organoids. Representative image with DsRED‐expressing normal organoids (red), green fluorescent protein (GFP)‐expressing EGFRvIII‐positive tumor organoids (green), and anti‐CD45‐allophycocyanin (APC)‐labeled EGFRvIII‐CAR NK‐92 cells (magenta) at 0 and 10 h of coculture. Scale bar: 200 µm (Reproduced with permission.^[^ ^116^ ^]^ Copyright 2019, EMBO). Cartoon images were created using BioRender.com.

Direct real‐time imaging assays are routinely used in cell biology to observe cell–cell interactions and as a platform for drug screening.^[^ ^113^, ^114^ ^]^ In many previous studies, tumor cell viability has been measured by seeding cancer cells on well plates or on glass coated with adhesion molecules such as gelatin, Matrigel, and fibronectin. In general, drug screening experiments are performed by treating monolayer‐cultured cancer cells with a drug and comparing the cancer cells killed at different time points. Many studies have been performed to observe apoptotic processes in cancer cells using live cell imaging.^[^ ^115^ ^]^ Images were acquired every 5 min for ≈6–12 h, and the interaction between NK‐92 cells and cancer cells was observed in real‐time (**Figure** **3**A). Direct imaging revealed that Nogo receptor 1 ((NgR1), an NK cell inhibitory receptor) plays a crucial role in regulating NK cell‐mediated killing by disrupting IS formation. By inhibiting NgR1, they demonstrated enhanced stability of NK‐target cell contacts by regulating NK‐cell F‐actin dynamics during IS formation. Direct imaging serves as a valuable quantitative analysis tool to ensure reliable and reproducible results for cell–cell interactions.

Although direct real‐time imaging assays have provided insights into cellular and molecular biology, they do not reflect the complex human TME. 3D cell culture models can reproduce the 3D tissue architecture of humans and spatially and temporally recapitulate the morphogenetic events resulting from human stem cell differentiation. Within tissues, cells interact with other cells and the ECM in a 3D communication network that is critical for tissue development, function, and survival. However, traditional 2D in vitro assays cannot mimic the dynamics encountered by immune cells that kill cancer cells within solid tumors. In addition, these methods neglect the obvious 3D forms of solid tumors and cancer cell clusters required for metastasis. Recently, many chips have been developed to observe the interactions between tumor spheroids and immune cells in a 3D tumor‐immune microenvironment.^[^ ^73^ ^]^ A 3D patient‐derived colon organoid model was developed to study the cytotoxicity of the CAR in NK‐92 cells^[^ ^116^ ^]^ (Figure 3B). Using a firefly luciferase reporter, which is described as a convenient readout of viable cells following cytotoxicity against a 2D cell line, luciferase/GFP expression was introduced into the organoids.^[^ ^91^ ^]^ Luciferase/GFP‐expressing organoids were inserted into a thin layer of Matrigel or suspension, followed by the addition of NK‐92 cells. The results showed that NK cells migrated readily to the surface but were unable to infiltrate the dense ECM. After 8 h, incubation only on the Matrigel‐coated layer and not on the suspension resulted in significant CAR‐induced lysis. They used this assay to test the killing ability of CAR NK‐92 cells toward patient‐derived normal and tumor organoids, as well as the antitumor efficacy of various types of CAR NK‐92 cells.

The direct real‐time imaging assay is a straightforward methodology that enables direct visualization and observation of interactions between two distinct cell types. However, these models have several limitations. They are unable to evaluate the impact of an intricate TIME because of their simplified nature, and their applicability is restricted to specific target cancer cells.

Various efforts have been made to overcome these limitations using advanced microfabrication technologies. These microfabricated systems offer a higher degree of complexity and better representation of the TIME, allowing for a more comprehensive evaluation of the interactions between immune and cancer cells. By leveraging the microfabrication technology, researchers can explore the intricate interplay between various cell types in realistic and physiologically relevant contexts. This has opened new avenues for studying its dynamics and influence on tumor progression and immune responses.

### 4.2 Microwell Arrays Containing Immune Cells and Cancer Cells

Adoptive lymphocytes exhibit substantial heterogeneity, with the human repertoire expressing over 10^7^ different T‐cell receptors.^[^ ^117^ ^]^ This extensive diversity facilitates antigen‐specific immune responses against a wide range of potentially harmful antigens. Furthermore, even under identical conditions, lymphocytes exhibit functional heterogeneity at the single‐cell level during their expansion and differentiation. To address the need for single‐cell‐level functional analyses, microwells with dimensions ranging from 10–100 µm have emerged as powerful tools. These microwells facilitate the examination of various lymphocyte functions, including lytic granule release and cell–cell interactions at the cellular level.

Microwell arrays, encompassing both cancer cells and lymphocytes in confined regions, offer valuable long‐term live‐cell imaging. The simplicity of this process enables the fabrication of microwell arrays in various laboratories. While the initial microwell assays used polystyrene fabricated through injection molding,^[^ ^118^ ^]^ subsequent developments introduced a glass‐silicon microwell array. This design involves patterning a microwell array with upper and lower holes (stencil) on a silicon wafer using photolithography and subsequently attaching glass to the bottom silicon surface.^[^ ^119^, ^120^ ^]^ However, these approaches require expensive instruments, rendering them unsuitable for use in typical laboratory settings. To address this limitation, microwell arrays for lymphocyte analysis have predominantly employed soft lithography techniques. Among the various materials, poly (dimethylsiloxane) (PDMS) is commonly used in microwell arrays. PDMS offers excellent gas permeability, biocompatibility, and compatibility with other substances, making it suitable for long‐term cell cultures in microwell assays.^[^ ^121^, ^122^, ^123^, ^124^ ^]^ In addition, microwell arrays based on polyurethane acrylate cured by UV exposure within a short timeframe have been developed.^[^ ^125^, ^126^ ^]^

**Figure 4:**
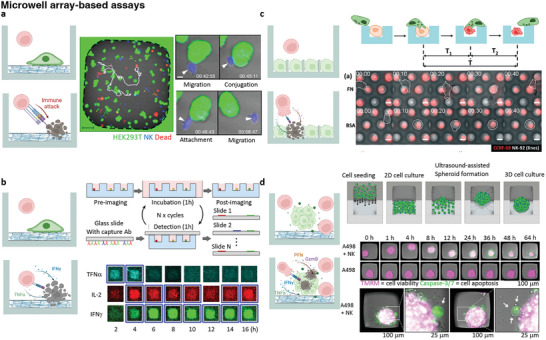
Microwell assay of evaluation of tumor cell killing and cytokine detection. A small amount of cancer cells and immune cells are seeded in the microwell array and their behavior was observed in real‐time. a) Microwell for imaging of immune cells to kill solid tumors; in each well, single cell level‐based antitumor efficacy evaluation analysis was performed (Reproduced with permission.^[^ ^128^ ^]^ Copyright 2015, American Association of Immunologists). b) Microwell for imaging of immune cell cytokine secretion; slide glass coated with the capture antibodies is placed in the microwell. When immune cells were incubated with tumor cells, the fluorescently labeled antibodies were detected and analyzed. (Reproduced with permission.^[^ ^131^ ^]^ Copyright 2012, National Academy of Sciences). c) Microwell for imaging of immune cells to kill hematological cancer cells; A microwell loaded with single hematological cancer cells (green) was fabricated. NK cells were seeded and interacted with cancer cells to kill them (Reproduced with permission.^[^ ^125^ ^]^ Copyright 2019, The Royal Society of Chemistry). d) Microwell for imaging of immune cells to kill tumor spheroids. A method for culturing 3D tumor spheroids by seeding cells in microwells and forming cell aggregates using ultrasonic‐based standing wave. Images were recorded every hour for 64 h by adding NK cells to time point “0 h.” tetramethyl rhodamine methyl ester (TMRM) (magenta) and caspase‐3/7 (green) were used as viability and apoptotic markers (Reproduced under the terms of the CC‐BY 4.0 license.^[^ ^139^ ^]^ Copyright 2022, Elsevier). Cartoon images were created using BioRender.com.

Microwell arrays are valuable tools for investigating single‐cell‐level cytotoxic functions, including the sequential steps of conjugating to the tumor, delivering lytic hits, killing the tumor, and subsequent detachment from the target (**Figure** **4**A).^[^ ^127^, ^128^ ^]^ These arrays have also facilitated the quantitative analysis of immune cell heterogeneity. Particularly noteworthy is the identification of a subpopulation of “serial killer” immune cells, capable of efficiently eliminating three or more cancer cells.^[^ ^129^ ^]^ Serial killer NK cells, in particular, exhibit enhanced cytotoxicity, delivering lytic hits more rapidly and releasing higher levels of perforin from each hit. This enables them to eliminate cancer cells at a considerably faster rate than NK cells, which target only one or two cancer cells.^[^ ^130^ ^]^ Integrating microengraving technology into microwell arrays allows the examination of correlations between cell and cell interactions and cytokine secretion by immune cells. By coexisting cytotoxic lymphocytes with cancer cells, researchers can observe the lysis of cancer cells and the subsequent secretion of IFN‐γ (Figure 4B), detect dead cells using SYTOX, and analyze secreted cytokines and chemokines using the microengraving technique. Captured antibody‐coated glass plates were placed in contact and incubated in a microwell array. The secreted proteins were bound to fluorescently labeled antibodies coated on a glass slide, and the secreted cytokines and chemokines were visualized. These events were then imaged and analyzed to establish correlations between secretion and killing events. Surprisingly, single‐cell‐based analysis revealed no direct correlation between cytolysis and INF‐γ secretion.^[^ ^121^, ^131^ ^]^ Liu et al. developed a novel microwell system to study hematological cancer cells suspended in the bloodstream. They employed optoelectronic tweezers to transfer cells into µ‐shaped microwells, providing a stable environment for observing NK cells targeting blood cancer cells.^[^ ^132^ ^]^ Doh et al. developed microwell arrays featuring specific antibodies immobilized on the microwell surface at regular distances. This arrangement enables the analysis of immune cell–blood cancer cell interactions, particularly focusing on the role of extracellular signals within the microwell array^[^ ^125^ ^]^ (Figure 4C).

Microwell arrays have also been employed in a range of tumor spheroid screening assays, providing a valuable platform for studying the effects of drugs on spheroids and organoids. In these assays, tumor cells were seeded on PDMS‐based microwell arrays at appropriate cell densities for spheroid culture and maintained for a specific period. Drug screening experiments were performed.^[^ ^133^, ^134^, ^135^ ^]^ Önfelt et al. utilized a microwell chip capable of supporting 2D or 3D cell culture with seeding densities ranging from 5 to 20 × 10^3^ cells. The design of the wells and the chosen material facilitated the formation of tumor spheroids using ultrasound.^[^ ^136^, ^137^, ^138^ ^]^ By utilizing the time‐averaged acoustic radiation force generated by standing ultrasonic waves, the cells rapidly aggregated at the center of the chamber^[^ ^139^ ^]^ (Figure 4D). This approach enabled the parallel production of numerous synthetic 3D solid tumors, allowing the investigation of NK cell behavior in terms of migration, docking, and killing of tumor spheroids. They characterized the tumor spheroids using high‐resolution and high‐throughput imaging techniques, which facilitated the observation of NK cell functionality. A microwell chip was used to evaluate the efficacy of NK cell killing by ovarian multicellular tumor spheroids in combination with drug therapy. Microwell‐based tumor spheroid experiments offer the advantage of precise size control over tumor spheroids, allowing researchers to observe the interactions between tumors and immune cells within the confined environment of microwells. Additionally, coating the PDMS micropillar array with capture antibodies facilitated high‐throughput detection of secreted cytokines, confirming the correlation between PD‐1 blockade and interleukin‐2 (IL‐2) secretion.^[^ ^140^ ^]^

In conclusion, microwell‐based cytotoxicity assays provide an effective high‐throughput screening platform for evaluating anticancer immunotherapies. This approach holds great promise in advancing our understanding of immunotherapy responses and in developing more effective cancer therapies. One limitation of microwell arrays is the difficulty of isolating single cells, which hampers the identification of the molecular characteristics of immune cells that display specific responses. Microwell arrays designed for cytotoxicity assays offer valuable insights into the functional heterogeneity of CLs at the single‐cell level, thereby contributing to the diagnosis and treatment of various immune‐related diseases. The fabrication process for these arrays is straightforward, allowing the development of new microwell assays tailored to specific experimental objectives. This versatility makes microwell arrays powerful tools for expediting the advancement of highly effective ex vivo‐engineered lymphocyte‐based therapies for cancer immunotherapy.

### 4.3 Droplet Microfluidics Generating Immune Cell–Cancer Cell Pairs

Droplet‐based microfluidics has emerged as a powerful high‐throughput tool for evaluating and investigating the functions and behaviors of immune cells. Droplet assays offer advantages such as better mixing, sorting, encapsulation, and sensing capabilities. The droplet microfluidic device consisted of two streams: one containing cells in the culture medium and the other containing oil for droplet formation. By flowing a cell‐containing stream, a droplet microfluidic device enables the coencapsulation of two cell types within individual droplets.^[^ ^141^ ^]^ Droplet platforms have been used in various applications including complex multistep biology,^[^ ^142^, ^143^ ^]^ chemical analysis,^[^ ^144^, ^145^ ^]^ DNA sequencing,^[^ ^146^, ^147^ ^]^ drug screening,^[^ ^148^, ^149^, ^150^ ^]^ and diagnostics.^[^ ^151^, ^152^, ^153^ ^]^

In 2005, Chiu et al. encapsulated single cells in picoliter‐volume aqueous droplets using a combination of optical trapping and microfluidic T‐junctions.^[^ ^154^ ^]^ Although these initial droplet studies exhibited low throughput and trapping efficiencies for single cells, they opened new possibilities for single‐cell studies in droplet microfluidics. Since then, continuous research has focused on improving efficiency and exploring methods to discard empty droplets through self‐sorting mechanisms. Control of the number of cells within each droplet was limited to a Poisson distribution. In another study, the probability of encapsulating only one cell in a droplet was constrained to 36.8%. Furthermore, the probability decreases to 13.5% when two different cell types are paired within a single droplet.^[^ ^155^ ^]^ Consequently, efforts have been made to standardize the geometry of droplet assays to address their limitations and efficiencies.

**Figure 5:**
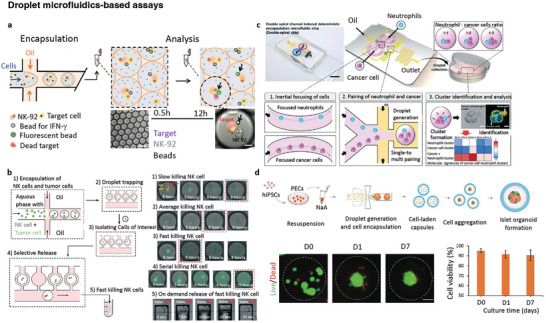
Microfluidic droplet devices encapsulating two types of cells. The cancer and immune cells are encapsulated within a droplet by microfluidic technology and their interactions are observed. a) Droplet assay for combinatorial evaluation of IFN‐γ‐release and NK cell cytotoxicity. Droplets containing NK‐92 cells, target cells, IFN‐γ‐sensing beads, and the detection antibody are generated. At different time points, the droplets were analyzed for target killing by NK cells and secretion of IFN‐γ by NK cells (Reproduced under the terms of the CC‐BY 4.0 license.^[^ ^158^ ^]^ Copyright 2020, Wiley‐VCH GmbH). b) Droplet assay to assess the heterogeneity of NK cells: 1) generated droplet encapsulated with NK and tumor cells. 2) Trapping droplets in docking sites. 3) Imaging the trapped droplets to determine the killing activity of NK cells. 4) Selectively releasing fast‐killing NK cells. 5) Collecting cells of interest (Reproduced with permission.^[^ ^161^ ^]^ Copyright 2022, The Royal Society of Chemistry). c) Droplet assay for cancer and neutrophil encapsulation: 1) inertial focusing of neutrophils and cancer cells via double‐spiral channel. 2) droplet generation and pairing of inertially focused cells with controlled ratios. 3) molecular analysis for identification of formed cancer–neutrophil clusters (Reproduced with permission.^[^ ^163^ ^]^ Copyright 2021, The Royal Society of Chemistry). d) Droplet assay for tumor spheroids formation; Flow chart of hybrid capsules used for the encapsulation of differentiated pancreatic endocrine cells from hiPSCs and the formation of islet organoids. Cell viability of islet organoids is evaluated at days 0, 1, and 7 of culture in capsules by live (green)/dead (red) staining and fluorescence quantitative analysis (Reproduced under the terms of the CC‐BY 4.0 license.^[^ ^166^ ^]^ Copyright 2020, Wiley‐VCH Verlag GmbH and Co. KGaA, Weinheim).

Building on the high efficiency of single‐cell droplet production, Cooper et al. effectively utilized it in their study. Using a fluorescence‐based technique, they closely monitored the binding of intracellular proteins to beads within the droplets. This enabled accurate measurement of the levels of two distinct intracellular proteins, Hras‐mCitrine in HEK‐293 cells and Actin‐eGFP in MCF‐7 cells. The study involved analyzing numerous individual droplets to ensure reliable and robust data, which were then quantified for further analysis.^[^ ^156^ ^]^ Furthermore, Huck et al. presented a microfluidic system capable of detecting cytokine secretions, such as IL‐2, IFN‐γ, and tumor necrosis factor‐alpha, from single T‐cells within droplets.^[^ ^157^ ^]^ To detect single‐cell‐secreted antibodies, fluorescent‐labeled detection antibodies and microdroplets coupled with capture antibodies were encapsulated in individual cells. Secreted substances were captured on the surface of the microdroplets, enabling the detection of localized fluorescent signals. Spatz et al. employed antibody‐functionalized polystyrene beads to quantitatively assess IFN‐ɣ‐secretion from NK cells encapsulated alone and coencapsulated with cancer cell targets (**Figure** **5**A). Droplets were collected at different time points (0.5, 3, 6, and 12 h) and placed in specially designed observation chambers. The release of IFN‐ɣ and NK cell cytotoxicity were subsequently analyzed using confocal fluorescence microscopy. Surprisingly, the results of this analysis revealed no substantial correlation between the occurrence of NK cell death events and IFN‐ɣ secretion.^[^ ^158^ ^]^

Microfluidic devices that compartmentalize cells into picoliter droplets have become crucial for 3D cell culture in vitro and closely mimic the physiological microenvironments of tissues and organs. Over the past decade, several groups have used droplet assays to analyze the behavior and function of immune responses. Konry et al. described a droplet microfluidics‐based system for monitoring interactions between live T cells and DCs. They observed cytoskeletal remodeling and microtubule polymerization in DCs at the IS formed within coencapsulated droplets. They also developed a system for monitoring calcium signaling in T cells upon contact with DCs.^[^ ^159^ ^]^ Based on these studies, droplet‐based cytotoxicity assays have been recognized as suitable models for evaluating the interactions between hematological cancer and immune cells. They encapsulated B‐cell non‐Hodgkin lymphoma and NK cells within droplets to evaluate the function of NK cells through quantitative assessments, such as the time required for killing and contact frequency.^[^ ^160^ ^]^ In their experimental setup, they encapsulated both NK cells and tumors.^[^ ^161^ ^]^ After rapid cell death (≈4 h), the device selectively released specific droplets, allowing the collection of NK cells. This unique capability enabled the recollection of NK cells that were still alive and active, demonstrating the ability of the platform to identify and rapidly retrieve the effector cells of interest while remaining intact and viable (Figure 5B). Zhao et al. also employed droplet assays to encapsulate T and target cells. They compared the differences in activation between specific and nonspecific T cell receptor‐engineered T (TCR‐T) cells by encapsulating T cells and cancer cells within droplets. As both cell types can be suspended within the droplets, stable measurements are possible over an extended duration. T‐cells were treated with a retroviral vector encoding a specific TCR gene and a lentiviral vector encoding the GFP reporter gene. This droplet‐based assay enables the screening of specific TCR‐T cells that recognize target tumor antigens. Nonspecific TCR‐T cells do not recognize target cells expressing the antigen, whereas specific TCR‐T cells are activated upon recognition of their cognate antigen, triggering the expression of eGFP.^[^ ^162^ ^]^ To selectively isolate the droplets of interest for molecular characterization, they developed an inverted float droplet array (iFDA) that allowed individual droplet monitoring. A UV laser at a wavelength of 355 nm was used to fractionate the droplets. Irradiation‐induced cavitation in the target wells creates air bubbles that facilitate droplet sorting in an iFDA system. After sorting, single‐cell reverse transcription polymerase chain reaction (PCR) was performed using universal TCR primers, and DNA from the PCR products was purified.

In addition to observing the direct contact of CLs with target cells, Jung et al. used a droplet assay to recapitulate circulating tumor cell‐neutrophil clusters (Figure 5C). They utilized the physical properties of a double spiral channel to create intricate pairs of neutrophils and cancer cells within droplets. This was accomplished through both single‐ and multiple‐encapsulation processes, enabling the formation of sophisticated clusters with various ratios (ranging from 1:1 to 1:3) of neutrophils to cancer cells. The biochemical characteristics of these clusters were determined based on E‐cadherin expression, and the interaction between cancer cells and neutrophils was assessed using vascular cell adhesion molecule 1. mRNA sequencing was employed to gain further insights into the molecular signatures involved in cell‐to‐cell associations.^[^ ^163^ ^]^ The deterministic encapsulation technique allowed precise control over the number of cells in each cluster, facilitating the generation of cancer cell‐neutrophil clusters of different sizes. This approach offers a valuable platform for studying the impact of metastatic seeding on cluster size. Furthermore, the cancer cell‐neutrophil clusters exhibited a hybrid epithelial/mesenchymal phenotype, corroborating the formation of a hybrid phenotype within the clusters.

Compared with single cells within droplets, spheroids or organoids with higher cell numbers require larger droplet volumes to accommodate an increased quantity of nutrients, growth factors, and byproducts essential for maintaining their viability and functionality^[^ ^164^, ^165^, ^166^ ^]^ (Figure 5D). Droplet microfluidics offers a solution that incorporates hydrogel scaffolds to encapsulate stable tumor spheroids and organoids. These hydrogels promote complex cell–matrix and cell–cell interactions in 3D cellular models. Various ECM‐derived materials such as collagen, Matrigel, and fibrin have been utilized as 3D substrates for culturing tumor spheroids and organoids.^[^ ^167^, ^168^ ^]^ Subsequently, these tumor spheroids or organoids can be treated with drugs and their responses can be observed.

Droplet microfluidic assays offer unique advantages over other devices because they enable cell pairing without the need for complex trapping structures. Furthermore, they provide a microenvironment that sustains cellular activity for an extended period. The isolation of specific droplets for analysis was straightforward. By merging droplets, droplet microfluidics allows the targeted insertion of a fluorescent probe at a desired time point to measure cytokine secretion from cells at specific intervals. However, droplet microfluidics may have a lower resolution than other assays, and a considerable number of droplets may be discarded based on the Poisson distribution. Therefore, additional monitoring assays are often required to obtain clear images for accurate analyses. Moreover, this model is more suitable for studying the interactions between floating cells within droplets and may not be an ideal choice for experiments involving solid tumors because it does not fully replicate the TIME.

### 4.4 Microfluidics Platforms Mimicking TIME

**Figure 6:**
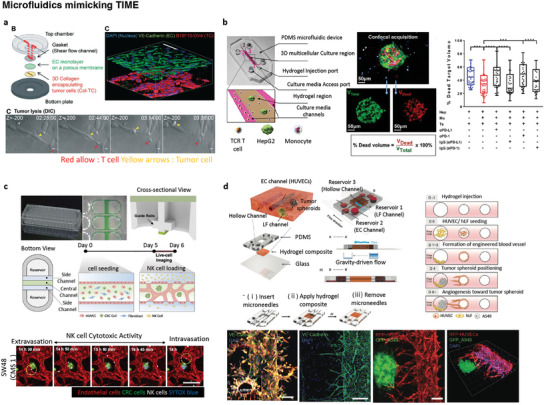
Representative 3D microphysiological systems reflecting the complex TIME. a) Multilayered blood vessel/tumor chip including 3D collagen gel containing tumor cells and endothelial cell monolayer cultured on a porous membrane. T cells are introduced through a flow channel on a porous membrane, and T cell infiltration and tumor killing are observed (Reproduced with permission.^[^ ^172^ ^]^ Copyright 2021, The Royal Society of Chemistry). b) A microfluidic device including three channels (a central gel channel and side medium channels). The engineered specific T cells were added into the device with GFP‐expressing HepG2‐Env aggregates embedded in the collagen gel. DRAQ7 was added in the culture media labeling dead cells red (Reproduced under the terms of the CC‐BY 4.0 license.^[^ ^175^ ^]^ Copyright 2018, Frontiers Media SA). c) Tumor vasculature model on the injection‐molded microfluidic platform. Cell configuration in the tumor. Vasculature models and perfusable blood vessel networks (red) formed within 5 d. Time‐lapse live‐cell images of NK cell (white) extravasation and cytotoxic activity. Scale bar: 200 µm (Reproduced under the terms of the CC‐BY 4.0 license.^[^ ^174^ ^]^ Copyright 2021, Frontiers Media SA). d) Microfluidics models based on hydrogels including perfusable and circular microchannels. Explosive view of the 3D vascularized lung cancer models. Immunofluorescence images of the engineered 3D tumor microvasculature. Scale bar: 200 µm (Reproduced under the terms of the CC‐BY 4.0 license.^[^ ^184^ ^]^ Copyright 2022, Wiley‐VCH GmbH).

Various microfluidics‐based tumor cytotoxicity models have been developed, providing advancements in this field.^[^ ^169^, ^170^, ^171^ ^]^ A multilayered blood/tumor tissue chip was developed to systematically explore immune cell infiltration into tumor tissues. This chip comprised a shear flow channel, a confluent endothelial cell monolayer cultured on a porous membrane, and a collagen gel block containing tumor cells (**Figure** **6**A).^[^ ^172^ ^]^ Although they used monolayered cultured blood vessels, they implemented a multilayered approach to mimic the TME and enabled the observation of immune cell behavior within the complex TME.

Recently, an organ‐on‐a‐chip was developed that incorporates blood vessels, as the process of locating and infiltrating the tumor stroma by immune cells is considered crucial. Jeon et al. made notable advances in the development of high‐throughput 3D cytotoxicity assays using a tumor‐vasculature‐on‐a‐chip system (Figure 6B). They utilized a rail‐based microfluidic design integrated within a single 96‐well plate, with the wells arranged in a 2×6 format. They successfully patterned HeLa cells encapsulated in collagen gel and examined the infiltration, migration, and cytotoxicity of NK‐92 cells. Their findings indicated that the presence of 3D ECM substantially impeded the migration of cytotoxic lymphocytes and their access to cancer cells, resulting in lower cytotoxicity compared to that in 2D assays. The dense ECM acted as a physical barrier, limiting the migration of cytotoxic cells; however, once in contact with the target cells, they demonstrated effective killing. The ECM has emerged as an important factor that influences both infiltration and cytotoxicity.^[^ ^173^ ^]^ In addition, live‐cell imaging of angiogenesis was performed using the same technology. They observed that primary NK cells migrated through blood vessels, specifically to regions where angiogenesis occurred, and subsequently targeted and killed tumor spheroids after blood vessel formation.^[^ ^174^ ^]^ Another example is the utilization of 3D microfluidics, which allows for the monitoring of immune cells as they are injected into a lateral channel and subsequently migrate into a central region filled with collagen, resembling “tissue” (Figure 6C). Wong et al. used a 3D microfluidic model to investigate and characterize the involvement of monocytes in T‐cell cancer immunotherapy. Their observations revealed that monocytes selectively suppress retrovirally transduced (Tdx) TCR‐T cell cytotoxicity via the PD‐L1/PD‐1 pathway, whereas the cytotoxicity of mRNA‐electroporated (EP) TCR‐T cells is unaffected by the presence of monocytes. Notably, under 2D coculture conditions, both Tdx and EP TCR‐T‐cell cytotoxicity were not hindered by the presence of monocytes.^[^ ^175^ ^]^ Borenstein et al. presented a multiplexed microfluidic system designed to evaluate immune–tumor interactions. Their microfluidic chip featured a V‐shaped postarrangement, allowing entrapment of tumor cells within a controlled flow field. This design facilitated the transport of oxygen and nutrients to trapped tumor cells over multiple days. Furthermore, the system enables real‐time high‐resolution imaging of interactions between immune cells and tumor tissues. This capability allows for the monitoring of tumor‐infiltrating lymphocyte‐mediated tumor killing and the assessment of tumor response to various treatment methods.^[^ ^176^ ^]^ Bertoletti et al. presented a 3D microfluidic model for the preclinical evaluation of TCR‐engineered T‐cells against solid tumors. Within the chip, a collagen gel solution containing HepG2 cells was injected into the dedicated gel region and TCR‐engineered T cells were introduced through the T cell injection port. Through experiments using these 3D models, they observed that the effect of oxygen levels on the lytic capacity of TCR T‐cells was consistent with the results of in vivo studies employing similar tumor‐bearing mouse models. Additionally, this study demonstrated that the 3D microfluidic model could uniquely detect the effects of changes in oxygen levels and the inflammatory environment on TCR T‐cell function, features that could not be identified using conventional 2D well‐based assays. This distinction highlights the importance of using 3D models to assess T‐cell behavior, particularly when assessing responses to various microenvironmental conditions and potential therapeutic interventions.^[^ ^177^ ^]^ Few advanced in vitro diagnostic systems that can effectively replicate patient‐specific tumor environments and have the potential to proactively assess immune‐based therapies in real time are available.

Finally, a microfluidic chip with circular vascularized perfusion microchannels was introduced to recapitulate and summarize the intricate complexity and interactions present in the TIME.^[^ ^178^, ^179^, ^180^, ^181^, ^182^ ^]^ Lam et al. evaluated the treatment of diffuse large B‐cell lymphoma (DLBCL).^[^ ^183^ ^]^ DLBCL is one of the most prevalent B‐cell lymphoma types. Although it is typically treated with a combination of chemotherapy and anti‐CD20 monoclonal antibody immunotherapy, ≈40% of the patients experience fatal outcomes. To investigate therapy resistance depending on the TIME subset, they recognized the need for a model that better replicated the intricate interactions between immune, cancer, and endothelial cells specific to DLBCL. Consequently, they developed a lymphoma‐on‐chip model that incorporated a tumor model based on hydrogels traversed by vascularized, perfusable, and round microchannels, which recapitulated the complexities and interactions of the TIME. The chip featured microvascular endothelial channels and hydrogel regions containing B cells, T cells, and macrophages. The endothelial channels penetrate the center of the hydrogel region. The efficacy of various treatment strategies was evaluated by injecting drugs or reagents into the endothelial channel and utilizing diffusion‐mediated transport to the hydrogel region. This chip represents a potent tool that enables researchers from diverse disciplines to study the TIME, with profound implications for drug delivery. Beebe et al. presented a microfluidic model using a similar chip design to study NK cell cytotoxicity and antibody‐dependent cellular cytotoxicity.^[^ ^179^ ^]^ Their chip incorporated a 3D hydrogel with two lateral lumens coated with endothelial cells, specifically human umbilical vein endothelial cells (HUVECs), to mimic the microvascular structures. Within the 3D hydrogel region, tumor spheroids comprising MCF7 and NK‐92 cells were observed. Antibodies were perfused through the lateral lumen to examine antibody kinetics and their impact on NK cell cytotoxicity. Notably, they observed that the penetration of antibodies into the spheroid was hindered by cell–cell junctions and that tumor cells could internalize antibodies within intracellular lipid vesicles. Furthermore, they confirmed that NK cells exhibited faster infiltration into tumor spheroids than antibodies and could migrate through tumor cell–cell junctions.^[^ ^180^ ^]^ Compared to conventional microfluidic models that require extensive expertise and sophisticated equipment for device development, this model offers the advantage of not requiring additional equipment such as pumps, sensors, and tubes. Kim et al. developed a chip with a similar design, in which angiogenesis was generated toward the tumor spheroid from the main blood vessel (Figure 6D). They successfully established conditions that facilitated well‐developed angiogenesis. Additionally, they demonstrated the rapid uptake of doxorubicin along newly formed blood vessels. They confirmed the ability of monocytes to infiltrate angiogenic blood vessels.^[^ ^184^ ^]^

Consequently, the use of ex vivo microfluidic devices that replicate the TIME has garnered considerable attention and will continue to be actively explored as a potent tool in preclinical trials. However, diverse multicompartment microfluidic chips must be developed that faithfully recapitulate the TIME, providing valuable insights into identifying challenges in immunotherapy and facilitating targeted improvements. The multicompartment microfluidic chip with blood vessels into which therapeutic agents can be delivered allows the research community to recapitulate actual transportation of various types of therapeutics, from small molecules to immune cells, into TIME, thus provide more realistic views of how different types of therapeutics influence each component of the TIME. Utilizing the multicompartment microfluidic chip can facilitate the assessment of whether cancer immunotherapeutic agents are effectively delivered into blood vessels and efficiently influenced tumors without affecting normal cells.^[^ ^185^ ^]^

### 4.5 Evaluation of Immune–Cancer Interactions through Image Processing

Either commercially available software, such as Imaris, or freely accessible open source software, such as ImageJ and Fiji, may be utilized for image processing. Image‐based cytotoxicity evaluation mainly rely on manual tracking of each cell using common image processing software. For instance, cell viability measurements involve continuous∖ tracking of Calcein fluorescence signal, which substantially decrease upon cell death due to the leakage of the dye.^[^ ^87^ ^]^ Similarly, when using death marker dyes such as PI or SYTOX, the emergence of a fluorescence signal is interpreted as an indication of cell death. Some research groups have defined the time point of cell death as the moment when drastic morphological change occurs in differential interference contrast images obtained through high‐resolution real‐time imaging.^[^ ^125^ ^]^

As the volume of data increases and the behavioral patterns requiring analysis become more complex, various automation algorithms are developed to facilitate analysis. The Onfelt group developed a partially automated analysis system that enabled the automated counting of live and dead cells within each microwell.^[^ ^120^ ^]^ With recent advancements in machine learning and deep learning technologies, there has been an increasing number of publications detailing the application of these techniques to cytotoxicity assays. The Konry group applied machine learning to analyze images for droplet cytotoxicity assays.^[^ ^186^ ^]^ The Park group developed a deep learning model to automatically analyze the immune synapses formed between CAR‐T cells and cancer cells.^[^ ^187^ ^]^

For image‐based analysis of 3D TIME models, more sophisticated algorithms are required to accurately extract diverse information. Parlato et al. extensively discussed image processing algorithms for 3D models, such as masking, boundary/feature extraction, and tracking, in their tutorial review article.^[^ ^188^ ^]^ For example, thresholding and boundary extraction algorithms were devised to quantitatively analyze immune cell infiltration into tumor tissues and tumor viability.^[^ ^176^ ^]^ In addition, feature extraction and tracking algorithms were employed to automatically analyze immune cell migration toward tumors.^[^ ^47^, ^189^ ^]^

These technological breakthroughs in image processing are essential for imaging‐based cytotoxicity assays since it enables high‐throughput data analysis with minimal human errors.

## 5 Tumor Organoid‐Based Systems for Evaluating Cancer Immunotherapy

**Figure 7:**
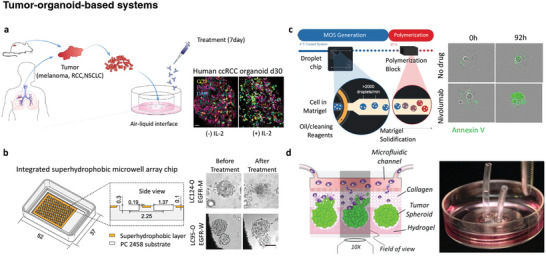
Representative image‐based tumor organoid systems. a) Air‐liquid interface method protocol. CD3+ TIL IF staining in d30 ccRCC PDO IL‐2 (100 IU mL^−1^). Scale bar: 20 µm (Reproduced with permission.^[^ ^192^ ^]^ Copyright 2018, Elsevier). b) Microwell array chip enabling observation of tumoroids. Bright‐field image of 1‐week drug treatment sensitivity measurements for tumoroids (Reproduced under the terms of the CC‐BY 4.0 license.^[^ ^197^ ^]^ Copyright 2021, Springer Nature). c) Droplet emulsion microfluidics to generate micro‐organospheres from low‐volume patient tissue. Representative image demonstrated gefitinib induces cell apoptosis. Scale bar: 100 µm (Reproduced with permission.^[^ ^199^ ^]^ Copyright 2022, Elsevier). d) Microfluidics models including gel port and media ports. Acridine orange (AO) and propidium iodide (PI) staining of MC38 on day 5 of ex vivo culture for anti‐PD‐1 (10 µg mL^−1^). Scale bar: 200 µm (Reproduced under the terms of the CC‐BY 4.0 license.^[^ ^203^ ^]^ Copyright 2021, IOP publishing Ltd).

The extensive heterogeneity observed in various TIME cell types poses considerable challenges for the development of effective therapeutic strategies.^[^ ^190^, ^191^ ^]^ In previous sections, we introduced various cytotoxicity models to evaluate the efficacy of immunotherapy. However, current 2D or 3D models do not fully reflect the interactions between tumors and immune cells in the TIME. Alternatively, the 3D tumoroid model has become increasingly necessary because it can reflect various factors of the TIME, such as the genetic background of patients, heterogeneity in patient solid tumors, and immune suppressive characteristics. In this section, we describe specific instances in which various cancer immunotherapies have been evaluated using tumor organoids (**Figure** **7**). The cancer immunotherapeutic approaches under scrutiny include adoptive cell therapy, immune checkpoint inhibitors, cancer vaccines, oncolytic virus therapy, and cytokine therapy.

### 5.1 Direct Real‐Time Imaging Assay for Assessing Immune Cell–Tumor Organoid Interactions

Patient‐derived organoids (PDOs) were cultured using the air–liquid interface (ALI) method, which not only recapitulates the epithelial and stromal compartments of the tumor, but also incorporates native immune cells, including T‐, B‐, and NK‐cells, and macrophages within the organoid structure. Subsequently, the tumor organoids were treated with anti‐PD‐1 and anti‐PD‐L1 agents, and the mRNA expression levels of crucial markers (granzyme B, perforin 1, and IFN‐γ) associated with tumor‐infiltrating T cell proliferation and activation were meticulously assessed. These results unequivocally demonstrate that this ex vivo culture approach successfully retains the intricate immune characteristics of tumors, thus presenting a valuable model for screening immune checkpoint blockade therapies.^[^ ^192^ ^]^

The PDO model using the ALI culture method lacks the infiltration of immune cells in the blood circulation into tumors, which is crucial for cancer immunotherapy.^[^ ^193^ ^]^ Due to this limitation, it cannot be deemed an ideal platform for comprehensively evaluating anticancer immunotherapies that stimulate the recruitment of immune cells from the bloodstream to dynamically change the TIME. To overcome this limitation, several research groups have added immune cells and cocultured them with PDOs. For instance, Courau et al. used cell line‐ and patient‐derived colorectal cancer organoids cocultured with allogeneic NK and T cells isolated from PBMCs. The cocultured immune cells swiftly infiltrated the tumor organoids and induced their destruction. Using this approach, they confirmed the potential of anti‐major histocompatibility complex (MHC) class I chain‐related protein A (MICA)and MHC class I chain‐related protein B (MICB) as an antitumor therapeutic agent by disrupting the NKG2D‐MICA/B pathway, consequently enhancing NK cell infiltration and immune activation toward the tumor. Furthermore, they demonstrated the synergistic effects of anti‐MICA/B and anti‐NKG2A antibodies, which block CD8 + and NK + inhibitory receptors.^[^ ^194^ ^]^ Using a similar approach, Dijkstra et al. cocultured epithelial tumor organoids derived from patients with nonsmall cell lung cancer and colorectal cancer with peripheral blood lymphocytes. This coculture method enables the proliferation of tumor‐reactive T cells. Furthermore, they confirmed that these tumor‐reactive T cells did not affect allogeneic normal tissue‐derived organoids, demonstrating the potential of this method as a robust platform for evaluating T cell‐related cancer immunotherapy.^[^ ^195^ ^]^

### 5.2 Microwell Assay Containing Immune Cells and Tumor Organoids

Microwell assays are valuable tools for assessing the effects of antitumor immunotherapy on tumor organoids. These assays are highly effective in evaluating cancer immunotherapy, with several key advantages, such as the controlled size of tumor organoids, high‐throughput capabilities for long‐term studies, and ease of imaging and analysis.

Jeong et al. employed a concave microwell array to generate size‐controlled multicellular tumoroids for anticancer drug screening.^[^ ^196^ ^]^ To generate multicellular tumoroids, the bovine serum albumin (BSA) solution was removed from the microwell array and a mixture of A549 cells (a human lung carcinoma cell line), HUVECs, and MRC‐5 cells (a human lung fibroblast cell line) was gently plated on the array. After successful multicellular tumor generation, the group proceeded with drug screening experiments within concave microwell arrays, which allowed them to investigate the effects of anticancer drugs on tumoroids in a controlled and relevant microenvironment.

As an example of evaluating immunotherapy using microwell assays, Liu et al. demonstrated another important application of microwell assays. They successfully generated lung tumor organoids by culturing the PDOs in microwells for an extended period. Direct drug sensitivity tests were also conducted using microwell assays.^[^ ^197^ ^]^ These cases demonstrate that microwell assays are valuable tools for assessing the efficacy of antitumor treatments in tumoroid models.

### 5.3 Droplet Microfluidics Encapsulating Immune Cells and Tumor Organoids

Droplet assays, which have the advantages of microwells, are widely used in patient‐derived tumor organoid experiments, with the advantage of being able to freely incorporate an ECM, such as Matrigel, when encapsulating tumor organoids.

To investigate the high‐throughput generation of mammary tumor organoids, Ortega et al. collected tumor tissue samples (≈200 µm) from 12‐ to 14‐week‐old mouse mammary tumor virus‐polyoma middle tumor‐antigen (MMTV‐PyMT) mice which are genetically engineered mice that spontaneously develop breast cancer. Subsequently, tissue slices were encapsulated in a drop array using alginate microbeads as the encapsulation material. Within this controlled environment, alginate‐encapsulated tumor pieces were allowed to grow and develop for ≈1 week. Following the successful growth of breast tumor organoids, various experiments and analyses were performed. Phenotypic analysis was performed to study the characteristics and behavior of tumor organoids.^[^ ^198^ ^]^

An example of immunotherapy evaluation via droplet microfluidics is the extensive study of droplet analysis for patient‐specific treatments.^[^ ^199^ ^]^ Shaohua et al. presented an automated system that sequentially included organoid production, manipulation, and drug evaluation functions, and succeeded in producing organoids of the same size using a certain number of cells.^[^ ^200^ ^]^ By doing this, they aimed to achieve high‐throughput screening of cancer patients in one week, which is an important goal for personalized medicine. These cases demonstrate that the droplet assay is a suitable model for evaluating immunotherapeutic agents, leveraging the ability of the tumor‐type model to seamlessly integrate with the ECM.

### 5.4 Ex Vivo Microfluidic System Recapitulating Complex Tumor‐Immune Microenvironment

Finally, an ex vivo system was developed to incorporate the features of TIME and model the dynamic response to ICB. Jenkins et al. made important contributions to the field of cancer research by introducing a microfluidics‐based platform to evaluate both murine‐ and patient‐derived organotypic tumor spheroids and organoids. Their study successfully demonstrated that tumor‐derived spheroids, with sizes ranging between 40 and 100 µm, exhibited comparable fractions of T lymphocytes and bone marrow populations with those of in vivo tumors. In these experiments, tumor tissues obtained from rats and patients were carefully filtered and loaded onto the gel channel of a microfluidic device. They utilized the media channel of a microfluidic setup to observe the effects of PD‐1 blockade on tumor tissues. This approach allowed us to study the response of TME to PD‐1 blockade in a controlled and dynamic manner.^[^ ^201^, ^202^ ^]^

The use of microfabrication systems to observe tumor organoids is promising for advancing cancer research, particularly in the field of immunotherapy. However, maintaining organoid viability and function outside the human body can be challenging, limiting their widespread use in research and therapeutic development. Once these challenges are overcome, tumor organoid immunotherapy evaluation platforms offer several advantages. It can provide a more accurate representation of the TME, allowing the investigation of complex interactions between tumors and immune cells in a controlled microenvironment. This platform can also facilitate the screening of various immunotherapeutic agents and their effectiveness in patient‐specific tumor samples, potentially leading to personalized treatment approaches. Additionally, studying tumor organoids in microfabricated systems can reduce the reliance on animal models and increase experimental throughput, thereby making research more efficient and ethical.

## 6 Conclusions and Perspectives

The development of antitumor immunotherapeutic agents has necessitated the use of various platforms to evaluate treatment efficacy. Population evaluation assays, such as fluorescence intensity measurements, FACS, and mouse models, offer the advantage of observing the effects of treatments on tumors. However, a combination of live cell imaging and microfabrication techniques is required to evaluate the efficacy of immunotherapeutic agents using appropriate in vitro mimetics. Several microfabricated constructs have been developed to evaluate the effectiveness of antitumor agents in this context. Although microwell assays cannot fully replicate the complex ECM and vascularized TME, single‐cell assays allow the determination of cellular heterogeneity and the analysis of direct interactions between immune cells and spheroids. Another approach is the use of droplet assays, which allow single‐cell analysis and subsequent isolation of the desired droplets to investigate the molecular properties of immune cells. However, as these platforms do not replicate the vascularized TME, a microfluidic chip containing blood vessels is required to observe these interactions.

Because all models were image‐based methods using a microscope, cell viability was obtained by calculating the ratio of the total tumor and the dead marker (PI, SYTOX) signal. Additionally, through real‐time microscopy, the migration of immune cells can be tracked over time and immune‐mediated killing mechanisms can be confirmed. Within this system, all models can be applied to various cancer immunotherapies such as adoptive cell therapy, immune checkpoint inhibitors, and cancer vaccines. Additionally, the principles of the technologies developed in this review can be adapted in various ways and utilized to validate and enhance Cancer Immunotherapy.

In conclusion, the utilization of 3D chips that accurately mimic the complex ECM can potentially accelerate the development of in vitro‐engineered CL therapies, which have demonstrated remarkable success in cancer immunotherapy. The ongoing development of 3D immuno‐oncology organoid chips represents a promising tool for translational research and bridges the gap between preclinical and clinical studies in the field of tumor organoid diseases. By comparing the strengths and weaknesses of each assay, effective evaluation methods can guide treatment development and facilitate further progress in this field.

## Conflict of Interest

The authors declare no conflict of interest.
